# A postmortem study suggests a revision of the dual-hit hypothesis of Parkinson’s disease

**DOI:** 10.1038/s41531-022-00436-2

**Published:** 2022-11-30

**Authors:** Per Borghammer, Mie Kristine Just, Jacob Horsager, Casper Skjærbæk, Anna Raunio, Eloise H. Kok, Sara Savola, Shigeo Murayama, Yuko Saito, Liisa Myllykangas, Nathalie Van Den Berge

**Affiliations:** 1 Department of Nuclear Medicine and PET, Aarhus University Hospital, Aarhus, Denmark; 2 Department of Clinical Medicine, Aarhus University, Aarhus, Denmark; 3 Department of Pathology, University of Helsinki, and HUS Diagnostic Center, University Hospital, Helsinki, Finland; 4 Brain Bank for Neurodevelopmental, Neurological and Psychiatric Disorders, United Graduate School of Child Development, Osaka University, Osaka, Japan; 5 Brain Bank for Aging Research, Tokyo Metropolitan Geriatric Hospital and Institute of Gerontology, Tokyo, Japan

**Keywords:** Parkinson's disease, Neuroscience

## Abstract

The dual-hit hypothesis of Parkinson’s disease (PD) originally postulated that a neurotropic pathogen leads to formation of α-synuclein pathology in the olfactory bulb (OB) and dorsal motor nucleus of the vagus (DMV) and then invades the brain from these two entry points. Little work has been conducted to validate an important underlying premise for the dual-hit hypothesis, namely that the initial Lewy pathology does arise simultaneously in the OB and the enteric nervous system (ENS) plexuses and DMV at the earliest disease stage. We conducted a focused re-analysis of two postmortem datasets, which included large numbers of mild Lewy body disease (LBD) cases. We found that cases with α-synuclein pathology restricted to the peripheral autonomic nervous system and/or lower brainstem (early body-first LBD cases) very rarely had any OB pathology, suggesting that Lewy pathology commonly arises in the ENS without concomitant involvement of the OB. In contrast, cases with mild amygdala-predominant Lewy pathology (early brain-first LBD cases) nearly always showed OB pathology. This is compatible with the first pathology being triggered in the OB or amygdala followed by secondary spreading to connected structures, but without early involvement of the ENS or lower brainstem. These observations support that the pathologic process starts in *either* the olfactory bulb *or* the ENS, but rarely in the olfactory bulb and gut simultaneously. More studies on neuropathological datasets are warranted to reproduce these findings. The agreement between the revised single-hit hypothesis and the recently proposed brain-first vs. body-first model of LBD is discussed.

## Introduction

The dual-hit hypothesis of Parkinson’s disease (PD) originally suggested by Braak, Del Tredici, and Hawkes postulated that a neurotropic pathogen enters the brain *simultaneously* via two routes: a nasal, with anterograde progression to the temporal lobe, and a gastric, with retrograde spreading to the dorsal motor nucleus (DMV)^1,2^. Overall, this theory seems to be in general agreement with the known observations that hyposmia and autonomic symptoms appear before parkinsonism in some cases of PD^3^. The dual-hit hypothesis has been much debated and has inspired a large number of clinical and preclinical studies investigating possible spreading routes of pathogenic α-synuclein and trigger factors, which could initiate or aggravate the initial formation of Lewy pathology in these two origin sites^4–11^.

Nevertheless, very little work has been carried out to study and validate an important underlying premise for the dual-hit hypothesis, namely that the initial Lewy pathology in fact does arise *simultaneously* in the olfactory bulb (OB) and the enteric nervous system (ENS) plexuses and DMV at the earliest disease stage.

To this end, we conducted a focused re-analysis of two recently published postmortem datasets from Vantaa, Finland, and Tokyo, Japan. Both datasets included a large number of patients with mild incidental Lewy body disease (ILBD). Of note, we assume that mild ILBD is the asymptomatic preclinical stage seen years before more widespread Lewy body disease characteristic of clinical PD and dementia with Lewy bodies. This temporal relationship is also an underlying assumption in all major neuropathological staging systems of Lewy body disorders^12–15^.

**Fig. 1:**
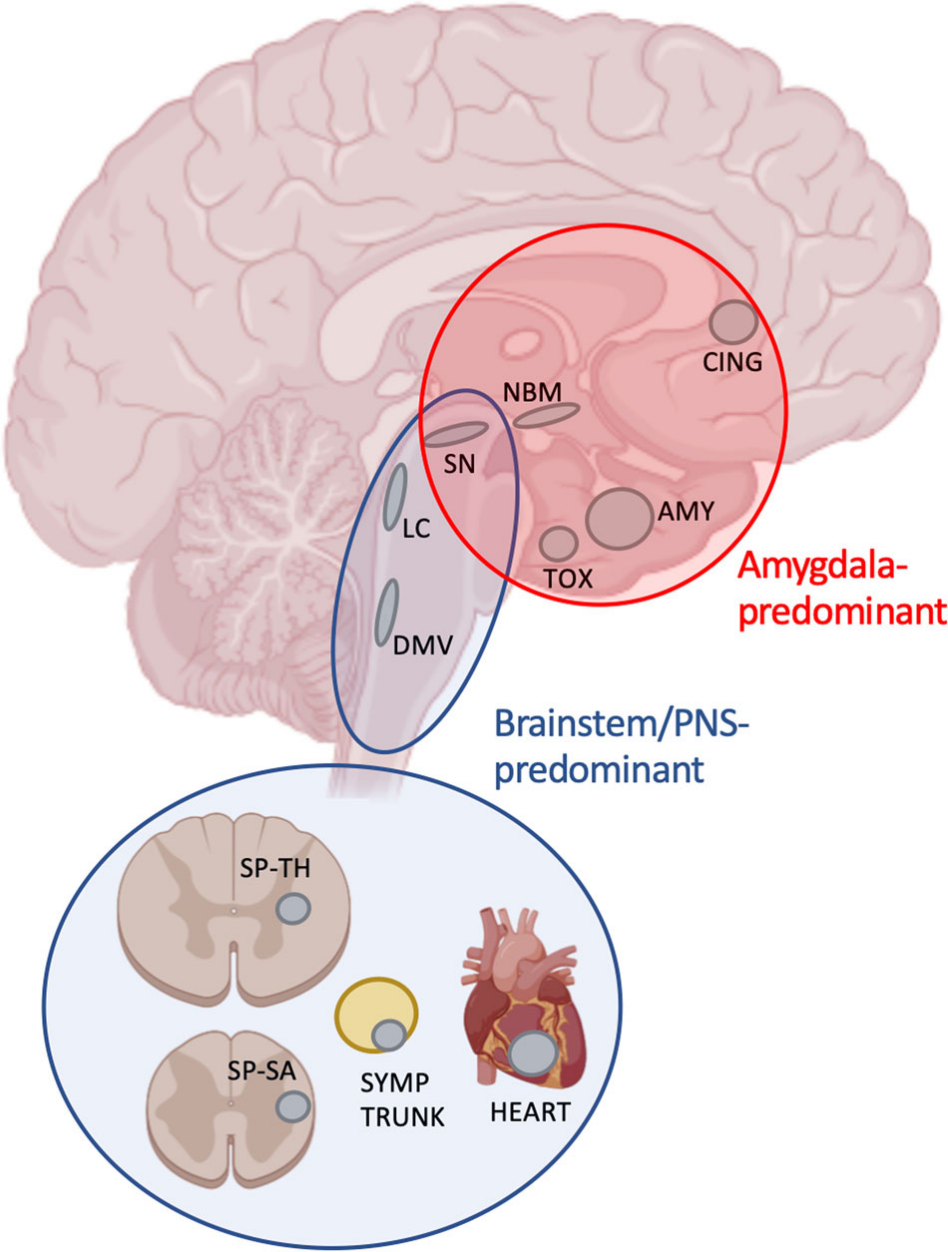
Schematic illustration of the important neuroanatomical structures investigated in this study. Amygdala-predominant cases had Lewy pathology in 1–3 structures predominantly within the red circle (in all cases including the amygdala), but limited or no pathology in brainstem or autonomic nervous system. In the brainstem-/PNS-predominant category (blue ellipse and circle), most cases had Lewy pathology in 1–3 structures in the brainstem (blue ellipse), except 9 cases with pathology restricted to the sympathetic trunk and/or heart (blue circle). AMY amygdala, CING cingulum, DMV dorsal motor nucleus of vagus, LC locus coeruleus, NBM nucleus basalis of Meynert, OB olfactory bulb, SN substantia nigra, SP-SA sacral spinal cord, SP-TH thoracic spinal cord, TOX transentorhinal cortex.

The two datasets contained 302 Lewy pathology-positive cases. In the main analysis, we included only ILBD cases with very mild pathology, defined as cases with pathology in 0 to 3 CNS regions (not including the OB itself). There were 80 such cases with mild ILBD and 71 (89%) of these could be divided unambiguously into two categories, those with amygdala-predominant Lewy pathology, and those with pathology restricted to the peripheral autonomic nervous system (PNS) and/or spinal cord and lower brainstem (Fig. 1). The third group comprised the remaining 9 cases (11%), which could not be assigned to the two primary categories. We then assessed the frequency of OB pathology in the two primary categories of ILBD cases to test whether OB pathology is universally present irrespective of the distribution of Lewy pathology elsewhere in the central nervous system (CNS) and PNS, which would be predicted by the dual-hit hypothesis. Finally, we assessed the frequency of Alzheimer’s disease (AD) co-pathology in the amygdala-predominant and brainstem/PNS-predominant groups.

## Results

### Olfactory bulb pathology

#### Vantaa dataset

Of the 124 Lewy-body positive cases in the Vantaa dataset, 27 were early cases with pathology in only 1–3 CNS locations (Table 1). Of these, 16 cases showed a brainstem/PNS-predominant distribution of Lewy pathology and only 2 (13%) of these cases had OB pathology. Five of the 27 cases showed an amygdala-predominant distribution of Lewy pathology and 100% of these cases had OB pathology. The remaining 6 cases had pathology only in the locus coeruleus (LC) and/or substantia nigra (SN), and 5 (83%) had OB-pathology.

**Table 1:** Vantaa dataset.

spSa	spTh	DMV	LC	SN	AMY	TOX	# cases	# OB pos.	% OB pos.
*EARLY AMYGDALA-PREDOMINANT CASES*									
					x		2	2	100
				x	x		1	1	100
		x			x		1	1	100
				x	x	x	1	1	100
		*TOTAL*					5	5	100
*EARLY BRAINSTEM/PNS-PREDOMINANT CASES*									
		x					4	0	0
	x	x					6	0	0
x		x					1	0	0
		x	x				1	0	0
x	x	x	x				2	1	50
	x	x			x		1	1	100
x	x	x	x	x			1	0	0
		*TOTAL*					16	2	13
*EARLY OTHER CASES*									
				x			2	1	50
			x	x			4	4	100
		*TOTAL*					6	5	83

The Vantaa dataset had 27 early-stage cases with minimal Lewy-type pathology ordered into amygdala-predominant, brainstem/PNS-predominant cases, and other cases categories. Each row in the table represents a distinct pattern of pathology shared by a number of cases (# cases). An “x” signifies the presence of Lewy-type pathology in individual anatomical structures. The three right-most columns list the total number of cases of each type, the number of OB-positive cases, and the percentage of OB-positive cases.

*spSa* sacral spinal cord, *spTh* thoracic spinal cord, *DMV* dorsal motor nucleus of vagus, *LC* locus coeruleus, *SN* substantia nigra, *AMY* amygdala, *TOX* transentorhinal cortex, *OB* olfactory bulb.

All advanced cases in the Vantaa dataset with Lewy pathology in 4 or more CNS regions showed OB pathology, irrespective of whether they had a brainstem/PNS- or amygdala-predominant distribution of pathology.

#### Tokyo dataset

Of the 178 Lewy-body positive cases in the Tokyo dataset, 53 were identified as early cases with pathology in only 0–3 CNS locations. Of the 53 cases, 9 were PNS-only cases with pathology restricted to the sympathetic ganglia and/or heart but no pathology in the CNS (Table 2). Twenty-three cases showed a brainstem/PNS-predominant distribution of Lewy pathology and only 2 (9%) of these 23 cases had OB pathology. Twenty-seven cases showed an amygdala-predominant distribution of Lewy pathology and 24 (89%) of these 27 cases had OB pathology. Three cases had pathology only in the LC and/or SN, and 2 (67%) of these had OB-pathology.

**Table 2:** Tokyo dataset.

SYMP	HE	ADR	DMV	LC	SN	NBM	AMY	TOX	CING	TEMP	# cases	# OB pos.	% OB pos.
*EARLY AMYGDALA-PREDOMINANT CASES*													
							x				11	9	82
x							x				2	2	100
x	x						x				1	1	100
							x	x			2	1	50
						x	x				1	1	100
				x			x				1	1	100
						x	x	x			1	1	100
							x	x	x		1	1	100
					x		x	x			1	1	100
					x	x	x				2	2	100
						x	x		x		1	1	100
				x		x	x				1	1	100
							x		x	x	1	1	100
			x		x		x				1	1	100
			*TOTAL*								27	24	89
*EARLY BRAINSTEM/PNS-PREDOMINANT CASES*													
	x										1	0	0
x											5	0	0
x	x										3	0	0
			x								1	0	0
x	x		x								1	0	0
x	x	x	x								2	0	0
x				x							1	0	0
			x	x							4	0	0
	x		x	x							1	0	0
			x	x			x				1	1	100
			x	x		x					1	0	0
x			x	x		x					1	0	0
x	x	x	x		x						1	1	100
			*TOTAL*								23	2	9
*EARLY OTHER CASES*													
				x							1	1	100
					x						1	1	100
				x	x						1	0	0
			*TOTAL*								3	2	67

The Tokyo dataset had 53 early-stage ILBD cases with minimal Lewy-type pathology ordered into amygdala-predominant, brainstem/PNS-predominant cases, and other cases categories. An “x” signifies the presence of Lewy-type pathology in individual anatomical structures. For further details including some abbreviations, see Table 1 legend.

*SYMP* sympathetic trunk, *HE* heart, *ADR* adrenal gland, *NBM* nucleus basalis of Meynert, *CING* anterior cingulum, *TEMP* temporal cortex.

Of the remaining 125 advanced cases with Lewy pathology in 4 or more CNS regions, 118/121 (98%) had OB pathology (in 4 cases, the OB was not examined). The three OB-negative cases had mild global pathology (GBS < 9) mainly in the brainstem and PNS, and thus also conformed to brainstem-/PNS-predominant cases.

#### Vantaa & Tokyo data combined

Combining the early cases from the Vantaa and Tokyo datasets yielded 39 brainstem-/PNS-predominant cases of whom only 4 (10%) showed OB pathology. In contrast, 29 of 32 (91%) amygdala-predominant cases had OB pathology. This difference was statistically highly significant (*p* < 10^−10^). The four brainstem-/PNS-predominant cases with OB pathology also showed pathology in the LC, SN, or amygdala, respectively (Tables 1 and 2). In contrast, OB pathology was not seen in a single one of the 24 cases, where Lewy pathology was restricted to the DMV, spinal cord, and/or peripheral autonomic system (Tables 1 and 2).

Finally, nine cases had pathology only in the LC and/or SN of which 7 (78%) had OB pathology. This frequency of OB pathology was significantly higher when compared to the brainstem-/PNS-predominant group (*p* = 0.0003), but not different from that of the amygdala-predominant group (*p* = 0.30).

### Alzheimer co-pathology

Table 3 summarizes the degree of neuritic plaque AD co-pathology in all (i.e., early to advanced) amygdala-predominant and in all brainstem/PNS-predominant cases from the two datasets. The cases are subdivided into four categories of global Lewy pathology burden scores (GBS): mild (1–10), moderate (11–20), severe (21–30), and very severe (31+).

**Table 3:** Neuritic plaque pathology in Lewy pathology GBS subgroups of the two datasets.

		*Global Lewy pathology Burden Score (GBS)*			
		1–10	11–20	21–30	31+
	CERAD	*Vantaa dataset*			
Amygdala	0-A (*n*)	1	0	0	0
	B-C (*n*)	9	11	9	9
	Total (*n*)	10	11	9	9
Brainstem/PNS	0-A (*n*)	8	10	6	6
	B-C (*n*)	8	6	11	18
	Total (*n*)	16	16	17	24
All Vantaa cases	0-A (*n*)	9	10	6	6
	B-C (*n*)	17	17	20	27
	Total (*n*)	26	27	26	33
	CERAD	*Tokyo dataset*			
Amygdala	0-A (*n*)	11	7	7	13
	B-C (*n*)	13	8	7	24
	Total (*n*)	24	15	14	37
Brainstem/PNS	0-A (*n*)	25	10	8	0
	B-C (*n*)	5	3	4	1
	Total (*n*)	30	13	12	1
All Tokyo cases	0-A (*n*)	36	17	15	13
	B-C (*n*)	18	11	11	25
	Total (*n*)	54	28	26	38

Global Burden Scores (GBS) signify the summed burden of Lewy pathology. The amygdala- and brainstem/PNS-predominant cases in both datasets are divided into GBS categories of global mild (1–10), moderate (11–20), severe (21–30), and very severe (31+) Lewy pathology. In each subgroup, the number of cases (*n*) with CERAD stage 0-A (none, mild) and B-C (moderate, severe) is listed. Note that the table does not contain the full samples from the two datasets, since 12 (10%) Vantaa cases and 32 (18%) Tokyo cases could not be unequivocally assigned to amygdala-predominant or brainstem/PNS-predominant categories, since they had equal amounts of pathology in the AMY + TOX and DMV + sympathetic structures used for categorization of cases.

In the Vantaa data, consisting of very old people, nearly all amygdala-predominant cases (i.e., 38 of 39) had CERAD stage B-C. In the brainstem/PNS-predominant groups, the mild (1–10) and moderate (11–20) GBS subgroups contained 50% and 38% CERAD stage B-C cases, respectively. The severe (21–30) and very severe (31+) subgroups contained 65% and 75% CERAD stage B-C cases, respectively.

In the Tokyo data, consisting of slightly younger cases, 50–54% of mild-to-severe (GBS 1–10, 11–20, 21–30) amygdala-predominant cases had CERAD stage B-C, and 65% of the very severe GBS cases had CERAD B-C. In the brainstem/PNS-predominant groups, the mild and moderate GBS group included 17–23% cases with CERAD stage B-C, and the severe GBS group included 33% cases with CERAD stage B-C. The very severe GBS group contained only 1 case. The declining number of brainstem/PNS-predominant cases at high GBS levels in the Tokyo dataset is caused by the neuropathological scoring system in this dataset, which tends to rate the amygdala higher than brainstem structures—as explained above in the methods section and discussed more below.

When studying each GBS category as a whole (i.e., combining amygdala- and brainstem/PNS-predominant cases into one group), in the Vantaa data, the frequency of CERAD B-C climbed from 65% (17/26) at the mild GBS level to 82% (27/33) at the very severe GBS level. In the Tokyo data, the frequency of CERAD B-C increased from 33% (18/54) at the mild GBS level to 66% (25/38) at the very severe GBS level.

## Discussion

The dual-hit hypothesis originally proposed that formation of pathogenic α-synuclein species starts simultaneously in both the OB and ENS plexuses and encroaches on the CNS in a two-pronged attack^2^. In the original postmortem study by Braak et al., serving as the basis for the dual-hit hypothesis, cases were included based on mandatory presence of Lewy pathology in the DMV^12,16^. Thus, all early-stage cases by design conformed to a brainstem-predominant distribution of Lewy pathology. Nevertheless, only 40% (8/20) of the Braak stage 1 cases in that study harbored Lewy-type pathology in the OB.

In the two newer datasets presented here, only 10% of early cases with mild brainstem/PNS-predominant pathology had Lewy pathology in the OB. These data do not support a dual-hit etiology. Thus, rather than the “*simultaneous OB and DMV”* scenario proposed by the original dual-hit hypothesis, the present data seem to instead support a “*single-hit OB or autonomic”* scenario.

It has often been argued that the dual-hit hypothesis receives support from the fact that PD patients develop constipation, REM sleep behavior disorder (RBD), and hyposmia prior to diagnosis^2^. However, less than half of PD patients develop constipation during the prodromal phase^17^, only approximately 1/3 of PD patients develop RBD prodromally^18,19^, and about 70% develop hyposmia before diagnosis^20–22^. These frequencies of prodromal symptoms also suggest that not all patients conform to the dual-hit hypothesis, but that there is more than one prodromal subtype of LBD, including a clinical brain-first subtype, which tend to develop parkinsonism before RBD and constipation emerges.

**Fig. 2:**
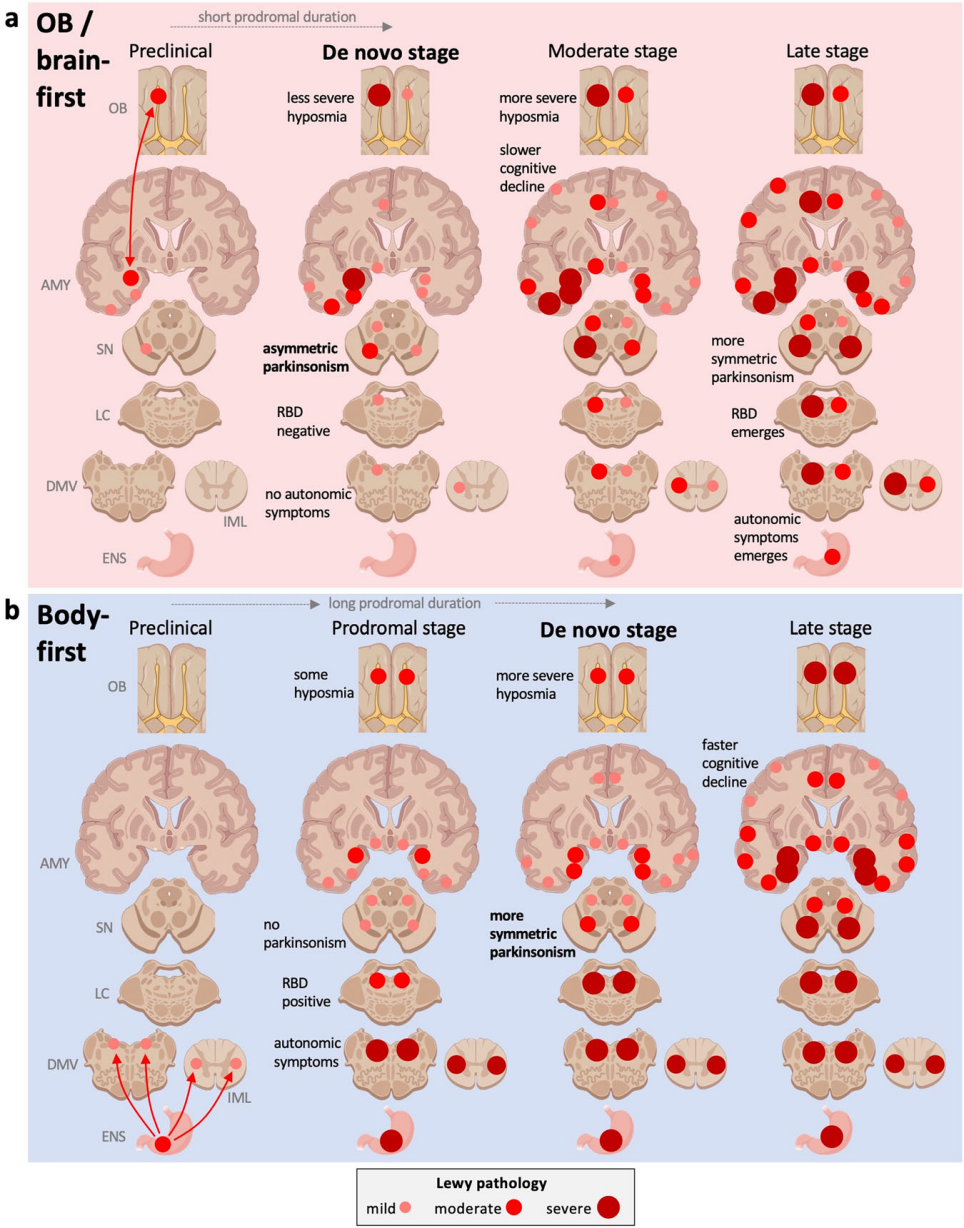
Single-hit hypothesis of prion-like propagation in Lewy body disorders (LBD). **a** *Brain-first/OB-first LBD*. Initial α-synuclein pathology arises in the OB or in the amygdala and spreads to closely connected structures. Due to the predominantly ipsilateral connections in one hemisphere, the pathology spreads initially to ipsilateral structures. At the time of diagnosis (de novo stage), the ipsilateral degeneration of the substantia nigra gives rise to asymmetric motor symptoms. The prodromal phase is relatively short, since pathology spreads rapidly from amygdala to ipsilateral SN. De novo patients are commonly RBD-negative, have few/no autonomic symptoms, and less frequent hyposmia, since one OB is often fairly intact, but also because the global CNS Lewy body burden is relatively low. The asymmetric distribution of α-synuclein persists into later disease stages, but at the end stage, both hemispheres are saturated (not shown). **b** *Body-first LBD*. The originating α-synuclein pathology arises in the ENS, propagates to the DMV and IML bilaterally due to overlapping autonomic innervation. The symmetric α-synuclein pathology propagates rostrally and leads to a more symmetric loss of nigrostriatal innervation and more symmetric motor symptoms. The prodromal phase is long, so there is more time for widespread dissemination of pathology. When parkinsonism emerges (de novo stage), the global burden of α-synuclein pathology is therefore higher in body-first LBD and there is more marked involvement of ascending, neuromodulatory brainstem nuclei, which may serve as efficient hubs for further widespread dissemination of α-synuclein pathology. De novo body-first patients are commonly RBD-positive, have autonomic symptoms, and more frequent hyposmia, due to bilateral OB involvement and also due to a higher global Lewy pathology burden throughout the CNS. The body-first patient is therefore at elevated risk of faster progression and accelerated dementia, measured from the time of onset of parkinsonism. See recent review on the SOC model for more details^23^. AMY amygdala, DMV dorsal motor nucleus of vagus, ENS enteric nervous system, IML intermediolateral cell column, LC locus coeruleus, OB olfactory bulb, SN substantia nigra.

A revised hypothesis might therefore postulate that external trigger factors (e.g., virus, toxins, or other insults) initiate the first α-synuclein misfolding and aggregation in only *one single location* in most patients (Fig. 2). Initial α-synuclein pathology in the OB mainly propagates unilaterally to the amygdala and closely connected structures, leading to an early amygdala-predominant distribution of Lewy pathology, as postulated by the *synuclein, origin and connectome* (SOC) model^23^. The SOC model assumes that clinical brain-first and body-first LBD subtypes reflect underlying amygdala- vs. brainstem/PNS-predominant distribution of Lewy pathology at early disease stages. Early amygdala-predominant (=brain-first) cases have little or no pathology in the lower brainstem and autonomic/enteric nervous system at early time points. Most of these *brain-first* patients do not develop RBD and autonomic symptoms until after parkinsonism has appeared. The prodromal phase is short due to early (unilateral) involvement of the SN via mono-synaptic connections between the central amygdala nucleus and SN pars compacta^24^.

In contrast, early brainstem/PNS-predominant distribution of Lewy pathology is caused by initial α-synuclein pathology in the ENS plexuses, which propagates to the DMV and, importantly, also via the sympathetic ganglia and trunk to the IML. Such body-first cases generally have no pathology in the OB or limbic system at the earliest preclinical time points, and they often develop autonomic symptoms and RBD years before diagnosis^7,23,25,26^. Body-first LBD is characterized by a long prodromal phase, which means that Lewy pathology is disseminated more widely at the time of diagnosis. Furthermore, bilateral PNS-to-CNS invasion of pathology, due to overlapping bilateral vagal and sympathetic innervation of the gastro-intestinal (GI) tract, causes a more bilateral disease progression in the brain of these patients. This wider disseminated pathology, including more bilateral involvement of cerebral structures, could explain the well-documented faster symptomatic progression and accelerated dementia in clinical body-first Lewy body disease^27–34^. Note that the majority of patients with Dementia with Lewy bodies are probably body-first, whereas only approximately 1/3 of PD patients show the body-first clinical phenotype^23,35–37^.

It is important to consider that there could be many different pathogenic trigger factors, and not just a single “smoking gun”. Some common factors may, in susceptible or just unlucky individuals, trigger α-synuclein pathology primarily or exclusively in either a nasal or in an ENS location, but rarely in both at the same time. Thus, some upper respiratory infections or inhaled toxins might trigger Lewy pathology primarily in the OB. In contrast, GI infections, GI inflammatory conditions, and some ingested toxins might trigger Lewy pathology primarily in the ENS. For instance, it has been shown that pesticide exposure is more common in PD cases with RBD and symmetric parkinsonism, i.e., a body-first subtype. This suggests that ingested pesticides may have a stronger propensity to trigger enteric pathology rather than OB pathology^29^.

In a minority of cases, the triggering event could initiate pathology in both the OB and enteric nervous plexuses, leading to a true dual-hit initiation of the disease. However, the present data suggest that such cases are relatively rare. Finally, in another minority of cases the first pathology may arise in the LC or SN before detectable pathology in the OB or autonomic structures appears, but such cases also seem to be relatively rare. These cases are not shown in the theoretical framework depicted in Fig. 2.

It may be helpful to consider α-synuclein pathobiology in the context of a cancer analogy. Implicitly, the original dual-hit hypothesis can be construed as postulating an almost *deterministic* process, i.e., that a pathogenic factor triggers formation of α-synuclein inclusions with *high probability*. In that hypothetical scenario, a pathogenic factor would commonly lead to simultaneous pathology in both the left and right OB and in the ENS. If this were true, one would expect that the OB, ENS, and DMV would show more or less simultaneous involvement in most ILBD cases at the earliest stages of Lewy pathology. But as reviewed here, this does not seem to be the case.

The revised single-hit hypothesis presented here suggests that the formation of the first pathogenic α-synuclein inclusions may be more of a *stochastic* process. Thus, the formation of seeding-competent prion-like α-synuclein species by an external trigger may be a very *low probability* event. This means that a trigger factor, e.g., a common virus infection, in most people may not lead to any sustained prion-like α-synuclein pathology at all, but in the rare cases when it does, it will most likely lead to only a single locus of pathology—perhaps in a single neuron (*neuron zero*). This locus could be in one OB (both OBs at the same time would be improbable) or in a small patch of the ENS plexuses—perhaps in a single parasympathetic motor neuron. The initial low-probability formation of prion-like species is then followed by a *high probability* secondary spreading from the originator locus. In most ILBD cases with minimal Lewy pathology, the distribution of pathology shows relatively stereotypical patterns and gradients, where the pathology is concentrated in one or a few heavily interconnected neurons. This distribution of pathology is more in line with an originally uni-focal disease with spreading to closely connected structures, more so than with a multi-focal disease, where pathology starts simultaneously in distant, unconnected structures, such as in the OB and enteric plexuses.

Thinking about α-synuclein pathobiology as a stochastic, low-probability process would make it analogous to cancer. Approximately 90% of all cancers have only one primary tumor, but most cancers metastasize sooner or later from this primary tumor^38^. Thus, the formation of the first cancer cell is stochastic, highly improbable, and therefore happens only in a single locus in most patients. In contrast, the subsequent metastatic propagation via available lymphogenic and hematogenic spreading routes is a high probability event, which in many cancers is inevitable given enough time. Since α-synuclein is a natively unfolded protein with countless different possible conformations, it seems plausible that the formation of a seeding-competent, prion-like conformation inside a single neuron could likewise be a rare, stochastic event^39^.

Olfactory dysfunction is important to the present discussion, since it develops in the majority of LBD patients, and frequently before diagnosis. Although counterintuitive, prodromal hyposmia seems to be more closely linked to the body-first subtype of LBD, which we assume has an underlying brainstem-/PNS-predominant profile of Lewy pathology. The majority of isolated RBD patients are hyposmic, and nearly all have lost the cardiac sympathetic innervation on [123I]MIBG scintigraphies, while their nigrostriatal dopaminergic innervation is still fairly intact^33,40^. In addition, another study reported that 71% of de novo PD patients with abnormal MIBG heart scintigraphy were hyposmic, while only 48% of de novo PD with normal MIBG were hyposmic—also suggesting that hyposmia is significantly more common in body-first LBD with pathological MIBG^20^.

The apparently closer association between hyposmia and body-first LBD can be explained in two ways. Recent postmortem studies have reported that hyposmia, measured before death, correlates with the *total burden* of CNS Lewy pathology, but show little or no correlation with the burden of Lewy pathology in the OB itself^41,42^. This means that hyposmia is a symptom of *widespread CNS Lewy pathology*, but not so much of pathology in the OB per se. This observation is in line with the idea that body-first LBD have more widespread Lewy pathology at diagnosis, including early involvement of both hemispheres. This equates to a higher total burden of CNS Lewy pathology, which could promote earlier development of hyposmia. Importantly, it must be remembered that the vast majority of olfaction tests is carried out with an identification test, where the subject must correctly name an odor. Such a test relies not only on OB function but also higher order information processing in olfactory cortex and memory. Thus, it may not be surprising that hyposmia, as measured with an identification test, correlates better with total burden of Lewy pathology than with pathology in the OB itself.

The second possible explanation, which may work in concert with the first explanation outlined above, relates to asymmetry. Briefly, in an OB- or brain-first case, the pathology would be triggered in one OB unilaterally, leaving the other OB initially intact. The neural connections in the mammalian CNS are highly lateralized and only ~1% of projections are commissural innervating the contralateral hemisphere. Thus, if the initial Lewy pathology locus is unilateral, the initial propagating pathology would remain mostly in the same hemisphere, causing quite asymmetric Lewy pathology in the CNS during early disease stages. Olfaction tests are nearly always performed on both nostrils at the same time, so even if one OB is dysfunctional, the patient will not appear hyposmic on such tests. This idea is supported by animal studies, where α-synuclein seeds are injected unilaterally into one OB, which leads to quite lateralized, asymmetric α-synuclein propagation in the hemisphere ipsilateral to injection^5,43^.

In contrast, body-first Lewy pathology spreads *bilaterally* to the CNS, since both the vagus and sympathetic nerves show a great deal of left-right overlap in their innervation fields of the ENS^23^. Thus, the initial Lewy pathology in the brainstem is already more bilateral from the onset, and therefore spreads more symmetrically. Approximately one-quarter of LC neurons directly innervate the OB, so once the LC is involved, the left and right OB may be directly seeded from the bilaterally affected LC^44^.

As also shown here, once Lewy pathology is widespread in the CNS (GBS > 9), all cases show OB pathology. The most parsimonious explanation seems to be that in brainstem/PNS-predominant cases, the OB is seeded as a secondary phenomenon from *within* the CNS—probably from lower brainstem structures such as the LC. This is corroborated by animal studies, where α-synuclein seeds injected into the gut or into the striatum propagates throughout the CNS and leads to marked OB pathology via centrifugal α-synuclein propagation^45,46^.

Of note, the second explanation implies that olfaction tests performed on each nostril separately might disclose unilateral hyposmia. However, two studies reported that hyposmia is *not* lateralized in early PD^47,48^, whereas another study did find unilateral hyposmia in early PD, which correlated with the most affected brain hemisphere^49^. Thus, it remains unclear whether unilateral hyposmia can occur in early PD.

Put together, hyposmia may arise earlier in body-first LBD, since (1) these patients may have a higher total burden of Lewy pathology at diagnosis, which has been shown to correlate with hyposmia, and (2) both OBs may be sufficiently involved earlier during the prodromal stage of body-first LBD compared to brain-first LBD. The first of these two explanations may be the most important.

Finally, we note that brain-first PD patients (without prodromal RBD) generally show more asymmetrical parkinsonism and asymmetrical dopaminergic loss on imaging, compared to RBD-positive body-first PD and DLB patients, who display on average much more symmetric dopaminergic loss on imaging^27,29,50,51^. These observations lend support to the fundamental idea that body-first LBD has greater involvement of both hemispheres at diagnosis compared to the more lateralized brain-first subtype.

Approximately 10% (9/80) of cases presented here showed very early pathology limited to the LC and/or SN (Tables 1 and 2). Of these, 78% (7/9) had OB pathology, which was similar to the frequency seen in the amygdala-predominant cases. Five of these 7 OB-positive cases had pathology in the LC, so their OB + LC pattern of pathology could be explained by the direct LC-to-OB connections^44^. A direct SN-to-OB tract has also been demonstrated in rats, which could account for the two remaining cases with OB + SN pathology^52^. Nevertheless, the high frequency of OB pathology in LC/SN-only cases remains to be explained.

Cases with amygdala-predominant profiles of Lewy pathology had higher frequencies of neuritic plaque co-pathology. This is in accordance with previous findings^53–55^. It has been suggested that amygdala-predominant Lewy pathology is a special type of pathology seen mainly in association with AD co-pathology^16^.

However, the Tokyo dataset suggests that although an amygdala-predominant profile is more commonly seen in association with neuritic plaques, approximately 50% of early amygdala-predominant cases showed little or no such AD co-pathology (CERAD 0-A). This frequency is in general agreement with previous studies investigating the amount of AD co-pathology in amygdala-predominant Lewy pathology. In the Vantaa data, nearly all amygdala-predominant cases showed CERAD B-C stages, which could be related to the extreme age (92 ± 4 years) of this population-based cohort of cases.

Regarding the brainstem/PNS-predominant cases, in the Vantaa data, the frequency of CERAD B-C stages increased from 40–50% at mild and moderate GBS levels to 65–75% at severe and very severe GBS levels (Table 3). In the Tokyo data, the frequency of CERAD B-C was much lower (17–23%) in brainstem/PNS-predominant cases. However, the Tokyo dataset is skewed at high GBS levels, where most cases are designated to the amygdala-predominant profile, since the AMY and TOX are commonly given a score of 4, whereas brainstem nuclei are never scored above 3^56^.

Importantly, when the cases were not divided into amygdala- and brainstem/PNS-predominant cases, but instead the CERAD B-C frequencies simply calculated in each GBS category, we find that the frequency of CERAD B-C increases significantly in association with increasing global Lewy pathology burden—from 65% to 82% in the Vantaa data, and from 35% to 59% in the Tokyo data (Table 3).

**Fig. 3:**
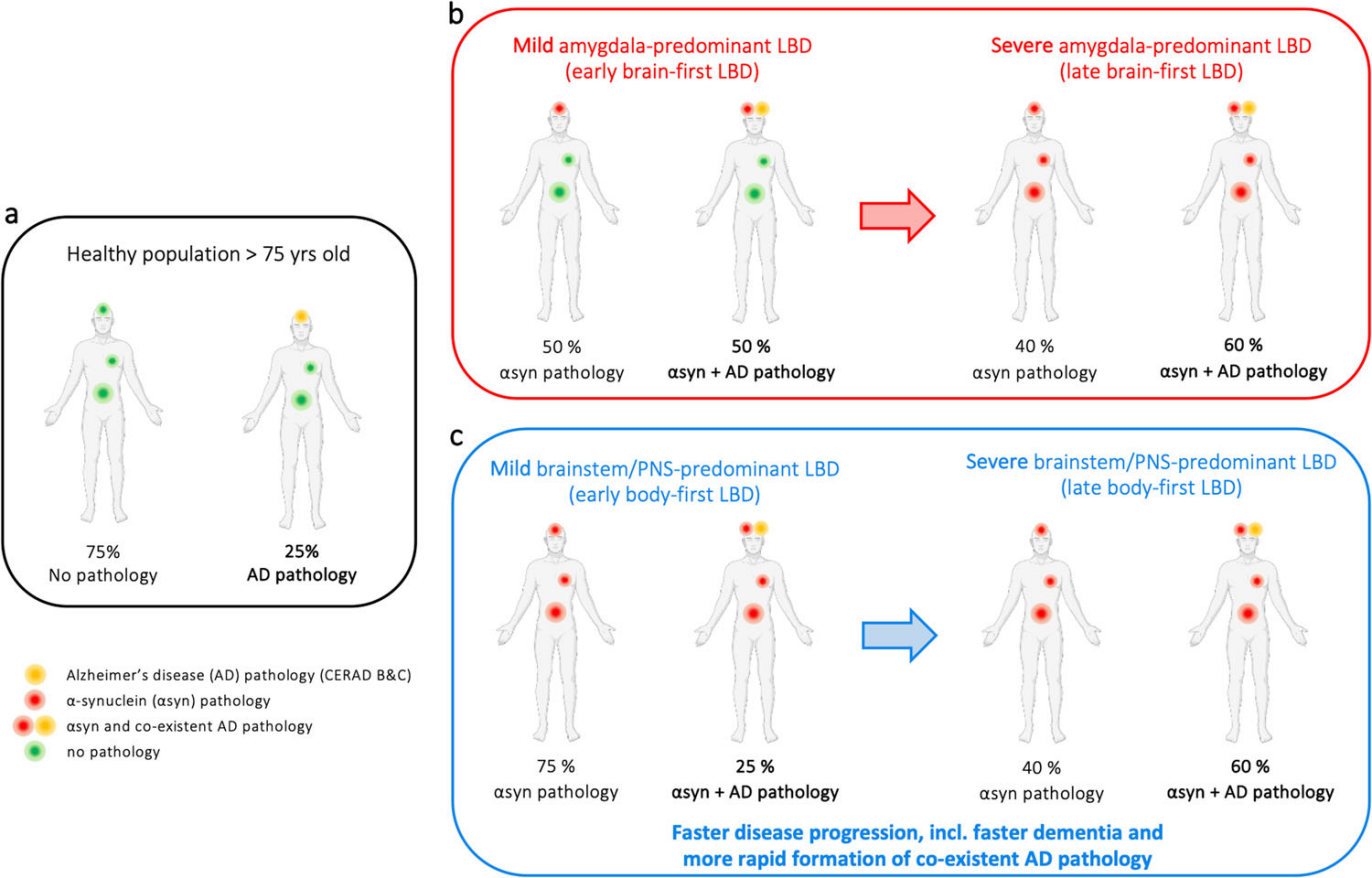
Alzheimer co-pathology in LBD. **a** Alzheimer (AD) co-pathology is common in the aged population. **b** Cases with early-stage amygdala-predominant LBD have more frequent AD co-pathology than expected for the age group, and the frequency of AD co-pathology increases further at late-stage disease. **c** Cases with early-stage brainstem/PNS-predominant LBD have AD co-pathology at the background level expected for the age group (same as in the black panel in **a**). However, cases with late-stage brainstem/PNS-predominant LBD display AD co-pathology almost as frequently as late-stage amygdala-predominant cases. This suggests that Lewy pathology promotes concomitant AD pathology, and this applies particularly to the brainstem/PNS-predominant group. [Note that the frequency estimates in the figure should be viewed as coarse approximations].

In summary, these observations lead to some tentative conclusions about AD co-pathology, graphically depicted in Fig. 3. First, amygdala-predominant Lewy pathology is more common in brains with concomitant AD pathology, but a large fraction of amygdala-predominant cases does not have more AD co-pathology compared to brainstem/PNS-predominant cases. This suggests that amygdala-predominant pathology can arise in the absence of AD co-pathology. However, it can be speculated that OB/amygdala-predominant Lewy pathology is more prone to appear in people who already have co-existing AD pathology.

Second, in early cases of brainstem/PNS-predominant cases, the frequency of neuritic plaque pathology does not seem to be above the background level expected for people at the particular age group studied^57^. Thus, it can be speculated that body-first Lewy pathology arises independently of co-existing AD pathology.

Third, as Lewy pathology accumulates in the brain (going from a low to a high global Lewy burden), the amount of neuritic plaque pathology also increases. This may be particularly true for cases with brainstem/PNS-predominant Lewy pathology, since these cases started at the background level of AD co-pathology. This suggests that accumulating Lewy pathology generally predisposes to secondary formation of amyloid-β (Aβ) pathology, perhaps via cross seeding mechanisms or simply because proteostatic mechanisms are overwhelmed and therefore unable to prevent accumulation of other types of protein pathologies^58,59^.

The third tentative conclusion mentioned above is important, since it potentially solves a paradox within LBD research: on the one hand, it is known that patients with a body-first subtype of LBD are at an increased risk of accelerated dementia. Prodromal RBD, constipation, symmetric dopamine loss, and pathological MIBG scintigraphies are all risk factors of dementia, and these symptoms and signs constitute the clinical body-first subtype^20,23,27,29,34^, assumed to correspond to the brainstem/PNS-predominant profile of Lewy pathology. On the other hand, it is well known that higher levels of AD co-pathology are seen in demented Lewy body patients at postmortem and also in vivo on amyloid PET scans^55,60^. However, the *early-stage* brainstem/PNS ILBD cases (=body-first) have less AD pathology than *early-stage* amygdala-predominant (=brain-first) ILBD cases, which constitutes the paradox. But as shown here, AD co-pathology seems to accumulate progressively in association with an increasing global burden of Lewy pathology, and this may be especially true in brainstem/PNS-predominant cases, which provides a solution to the apparent paradox.

In short, brainstem/PNS-predominant cases seems to start out with normal, background levels of AD co-pathology, but this particular subtype of Lewy pathology may be more aggressive, perhaps due to the more symmetrical, ascending CNS pathology as proposed by the SOC model^23^. This more aggressively propagating pathology may in turn predispose to more rapid induction of AD co-pathology. Therefore, end-stage body-first cases with severe levels of global Lewy pathology will on average also display high levels of AD co-pathology, even though they did not have particularly high levels of AD pathology when their brainstem/PNS-predominant Lewy pathology originally started.

This study has several limitations. The study was carried out on postmortem cases with very limited Lewy pathology. The assumption of this study was that such early-stage ILBD patients are representative of early preclinical stages of PD and DLB, but this has never been formally proved due to the cross-sectional nature of postmortem data.

Lewy-type pathology is studied with variable methodologies. Braak and colleagues used immunostaining against total α-synuclein^12^. Thus, it is possible that not all of the 40% OB-positive Braak stage 1 cases represent true α-synuclein pathology, but instead a kind of non-specific α-synuclein species. On the other hand, Braak et al. used thick microsections (100 µm). This may be a more efficient way to screen for even minimal amounts of OB pathology, which could easily be missed in thin (5–6 µm) sections typically used by other groups, potentially causing false-negative results in the OB. Importantly, the two datasets interrogated in the present study was based on more modern immunostaining techniques directed toward pathological species of α-synuclein^56,61^. Thus, these datasets may more accurately reflect the presence of true α-synuclein pathology in the OB.

For the moment, it cannot be concluded, whether the findings of very rare OB pathology in brainstem/PNS-predominant cases of the two recent datasets, or alternatively Braak’s finding of 40% OB pathology in stage 1 patients more accurately reflects the truth. Of note, animal studies investigating Braak’s gut-first hypothesis often observe that the OB is one of the first affected brain areas (soon after lower brainstem involvement) upon gut-initiation of disease by injection of artificial pathology into the gut plexuses^9^.

Future studies or re-analyses of other existing postmortem datasets are needed to resolve this issue. In general, more work is also needed to define criteria for what constitutes pathological α-synuclein—especially in non-brain structures. For instance, it has been shown that norovirus infection in children triggers an apparent α-synuclein accumulation in the ENS, which disappears after a few months^62^. It has also been shown that α-synuclein and Aβ42 inclusions can be seen in the OB of children and young adults, potentially due to air pollution^63^. This suggests that formation of aggregated protein pathologies may be a common response to various insults, but it remains unknown whether such pathologies are unspecific and self-limiting or, alternatively, whether they always constitute true precursors of neurodegenerative disease.

In summary, the data reviewed suggests that a *single-hit hypothesis of PD pathogenesis* may be more appropriate, and at the very least, is worthy of more investigation. We found that cases with Lewy pathology restricted to the peripheral autonomic nervous system and/or lower brainstem, very rarely shows olfactory bulb pathology during the earliest disease stages. This suggests that Lewy pathology is triggered in the GI tract or autonomic nervous system without concomitant involvement of the olfactory bulb. In contrast, cases with mild, early-stage amygdala-predominant pathology nearly always show olfactory bulb pathology. This could be compatible with an external pathogenic factor triggering initial pathology restricted to the OB, which then spreads rapidly to amygdala and connected structures, but without initial involvement of the ENS.

This revised single-hit hypothesis is also in better agreement with the recently proposed brain-first vs. body-first model of Lewy body disorders, which is itself based on well-documented differences in symptoms and signs in prodromal subtypes of LBD patients.

## Methods

### Datasets

Data from two postmortem datasets were reviewed in this study. Full details on these cohorts and immunohistochemical methods including antibodies and protocols were previously published^56,61^. All subjects provided written informed consent before enrolling into the two studies.

The Vantaa85+ population-based cohort consists of 304 postmortem cases (age at death 92 ± 4 years), of which 124 were Lewy-body positive in at least one of the screened regions of the CNS^61,64^. Immunostainings were conducted with the disease-associated α-synuclein antibody 5G4 on 5 µm thick tissue sections (clone 5G4, 1:1000, AJ Roboscreen GmbH, Leipzig, Germany or Merck KGaA, Darmstadt, Germany)^65^. Lewy pathology severity was assessed with a semi-quantitative system in all anatomical regions (0 = absent, 1 = slight, 2 = moderate, 3 = severe, 4 = very severe)^61^, which is very similar to the DLB consortium staging system^13^. This study included 10 CNS and 2 spinal cord anatomical regions, including the DMV, LC, SN, amygdala (AMY), transentorhinal cortex (TOX), OB, thoracic spinal cord (spTh) and sacral spinal cord (spSa). The remaining anatomical regions are not listed here, since these regions did not show Lewy pathology in any of the early-stage cases primarily interrogated in the present study. Fifteen cases in the Vantaa dataset had olfactory bulb-only α-synuclein pathology, and were not included in this study.

The dataset from the Tokyo Brain Bank for Ageing Research consists of 518 postmortem cases (mean age at death 83 ± 9 years), of which 178 were Lewy-body positive in at least one of the screened regions of the CNS or PNS^56^. Immunostainings were conducted with the anti-phosphorylated-α-synuclein antibody pSyn#64 on 6 µm thick tissue sections (pSyn#64, 1:20,000, FUJIFILM Wako Pure Chemical Corporation, Japan)^15^. Lewy pathology severity was assessed semi-quantitatively following the DLB consortium guidelines (0 = absent, 1 =slight, 2 = moderate, 3 = severe, 4 = very severe)^13^. This study included 10 CNS and 6 PNS anatomical regions, including the DMV, LC, SN, nucleus basalis of Meynert (NBM), AMY, TOX, anterior cingulum (CING), temporal cortex (TEMP), OB, sympathetic trunk ganglia (SYMP), adrenal gland (ADR), the myocardium (HE), and esophagus. The remaining regions are not mentioned, as they did not show pathology in any of the early-stage cases primarily interrogated in this study.

Of note, in the Vantaa and Tokyo datasets, the peripheral OB and the anterior olfactory nucleus were assessed individually. In the present paper, a case is designated *OB-positive*, if one or both of these two structures contained any Lewy pathology. Also, in both datasets the presence of Alzheimer’s disease (AD) co-pathology was assessed. Neuritic plaques were assessed according to the CERAD protocol^66^ and neurofibrillary tangles according to the protocol by Braak and Braak^67^.

### Case categories & olfactory bulb pathology

From the Vantaa and Tokyo datasets, we identified cases with minimal Lewy pathology. In the Vantaa data, cases with pathology restricted to 1, 2, or 3 CNS locations (not including spinal cord) were considered early and included. In the Tokyo dataset, cases with pathology in 0, 1, 2, or 3 CNS locations were included. The Tokyo dataset contained 9 cases with Lewy pathology restricted to the sympathetic ganglia and/or heart without any concomitant CNS pathology, i.e., cases with “PNS-only pathology”.

For each case, we calculated a simple metric to determine whether the magnitude of pathology was greatest in autonomic structures or in the limbic system. A case was categorized as *Brainstem/PNS-predominant*, when the average pathology in the sympathetic ganglia (Tokyo) or thoracic spinal cord (Vantaa) plus DMV was higher than the averaged pathology of the amygdala and transentorhinal cortex. Conversely, a case was defined as *amygdala-predominant* when the average pathology in the amygdala and transentorhinal cortex was higher than in the sympathetic ganglia or thoracic spinal cord and DMV. We have previously shown that this simple dichotomization algorithm is able to categorize the majority of cases with early-stage Lewy pathology into these two groups and we have shown that a blinded K-means cluster algorithm identifies nearly the same subjects^26,61^. A minority of cases were not classifiable, since they had isolated pathology in the LC and/or SN (termed *other cases*). The anatomical regions and brainstem/PNS- vs. amygdala-predominant categories are illustrated in Fig. 1.

The presence or absence of OB pathology in each of these early cases was recorded. All individual cases are listed in tables under the three main categories (brainstem/PNS, amygdala-predominant, other). The Vantaa and Tokyo data were then fused into one dataset, and simple statistical group-comparisons were carried out on the fused dataset with GraphPad PRISM (ver. 8) using Fisher’s exact test.

We also recorded the frequency of OB pathology in all remaining cases, i.e., advanced cases with more widespread pathology corresponding to 4 or more CNS regions. Finally, we calculated a global burden score (GBS) of Lewy pathology for each case by summing the Lewy pathology severity score of all examined regions. Four GBS categories were defined: mild global pathology (GBS 1–10), moderate global pathology (GBS 11–20), severe global pathology (GBS 21–30), very severe global pathology (GBS > 30).

Importantly, the semi-quantitative assessment of pathology used in these studies is not normalized to the maximal pathology value of each individual brain region. For example, pathology in brainstem regions incl. the DMV is very rarely scored as 4 (very severe), even in cases with severe, widespread Lewy pathology. In contrast, the amygdala is commonly assigned a score 3 (severe) or 4 (very severe), even in cases with limited global pathology. Thus, the semi-quantitative scoring introduces a bias and limits differentiation of brainstem/PNS- vs. amygdala-cases at mid-to-late disease stages. At late stages, most cases will be categorized as amygdala-predominant by the simple categorization algorithm we used. However, at early and moderate levels of global Lewy pathology (i.e., GBS 1–10 and GBS 11–20, respectively), most cases can be unequivocally categorized into brainstem/PNS- or amygdala-predominant groups. Since most cases with such limited Lewy pathology fall distinctly into these two categories, we assume that the majority of later stage cases developed from these two original starting profiles, even though brainstem/PNS- and amygdala-predominant profiles are less clear at later stages.

## Data Availability

The data that support the findings of this study are available from the corresponding author upon reasonable request, e.g., for the purpose of checking and reproducing the findings in the present study.
